# Effectiveness of Mechanical Horse-Riding Simulators on Postural Balance in Neurological Rehabilitation: Systematic Review and Meta-Analysis

**DOI:** 10.3390/ijerph17010165

**Published:** 2019-12-25

**Authors:** Juan G. Dominguez-Romero, Assumpta Molina-Aroca, Jose A. Moral-Munoz, Carlos Luque-Moreno, David Lucena-Anton

**Affiliations:** 1 Department of Physiotherapy, University of Osuna, 41640 Seville, Spain; j_gab_dom@hotmail.com; 2 Department of Nursing and Physiotherapy, University of Cadiz, 11009 Cadiz, Spain; joseantonio.moral@uca.es (J.A.M.-M.); carlos.luque@uca.es (C.L.-M.); david.lucena@uca.es (D.L.-A.); 3 Institute of Research and Innovation in Biomedical Sciences of the Province of Cadiz (INiBICA), University of Cadiz, 11009 Cadiz, Spain

**Keywords:** robotics, cerebral palsy, stroke, postural balance, neurological rehabilitation

## Abstract

Mechanical horse-riding simulators consist of a device that mimics the movement of a real horse, generating between 50 and 100 three-dimensional physical movements (forward and back, left and right, up and down). The main objective of this study is to analyze the effectiveness of mechanical horse-riding simulators to improve postural balance in subjects with neurological disorders. The search was conducted during January–March 2019 in PubMed, Physiotherapy Evidence Database (PEDro), Cochrane, Web of Science, CINAHL, and Scopus. The methodological quality of the studies was evaluated through the PEDro scale. A total of seven articles were included in this systematic review, of which four contributed information to the meta-analysis. Statistical analysis showed favorable results for balance in stroke patients, measured by the Berg Balance Scale (standardized mean difference (SMD) = 3.24; 95%; confidence interval (CI): 1.66–4.83). Not conclusive results were found in sitting postural balance, measured using the Gross Motor Function Measure-66 (GMFM-66) Sitting Dimension, in patients with cerebral palsy. Most studies have shown beneficial effects on postural balance compared with conventional physical therapy. However, due to the limited number of articles and their low methodological quality, no solid conclusions can be drawn about the effectiveness of this therapy.

## 1. Instruction

Postural control could be defined as the ability to maintain posture in space and has an intimate relationship with the sense of balance [1]. The ability to maintain posture is an important factor in carrying out activities of daily living and in achieving independent individual development [2]. Balance is achieved through the integration and coordination of multiple body systems (vestibular, visual, auditory, motor, and higher-level premotor systems) [3]. The maintenance of balance encompasses the acts of maintaining, reaching, or restoring the center of body mass in relation to the base of sustenance within the limits of stability [4,5]. In that way, the body will make different adjustments known as balance reactions [6]. These balance reactions are carried out through a complex process involving the sensory system, the central nervous system, and the skeletal muscle system [7]. Therefore, the sensory system (vestibular, visual, and somatosensory systems) provides the information necessary to maintain an upright posture. This information is integrated by the central nervous system that will send an adequate neuromuscular response to the musculoskeletal system to perform the required movements [8]. Other authors [9,10] consider that balance is the result of the integration of inputs with the body as a mechanical system that interacts with the nervous system in a constantly changing environment. Evidence has shown that deficits or limitations in the underlying systems impair balance [11]. Horak et al. [12] described six major components required to maintain postural control: biomechanical limitations, movement strategies, sensory strategies, orientation in space, dynamic control, and cognitive processing. This framework emphasizes the need for an independent evaluation of each component and individualized treatment. Sibley et al. [13] completed this proposal performing an in-depth study of the different components of balance and their functional definition.

Concerning the rehabilitation of postural balance, different methods can be found in the current literature analyzing the effects of those interventions in different groups of the population. In that way, vestibular rehabilitation [14], vibration [15], and multimodal exercise show benefits on balance in the elderly. Innovative methods such as virtual reality [16], complementary therapies such as mind–body movements [17], treadmill training [18], and peripheral somatosensory stimulation [19] have been shown to have different benefits on balance after stroke. For cerebral palsy, different techniques have been used to improve postural balance: whole-body vibration [20], exercise-based interventions [21], therapeutic horseback riding, and hippotherapy [22].

Mechanical horse-riding simulator (HRS) is a type of intervention based on hippotherapy, consisting of a robotic device with a dynamic saddle that imitates the movement of a horse. Hippotherapy can be defined as animal-assisted therapy that uses the movement of an equine as a therapeutic tool and an adjuvant in the rehabilitation of a patient [23]. This is an effective intervention to aid motor recovery in patients with disorders of the central nervous system, such as cerebral palsy (CP) [24], multiple sclerosis, spinal cord injury [23,25], stroke [23,25], as well as Down’s Syndrome [26,27] and rheumatic joint diseases [28]. Hippotherapy improves physiological aspects including balance, strength, coordination, muscle tone, range of motion, weight-bearing, posture [23], anteroposterior stability [29], vertical alignment [30], and gait [23,25]. In addition to influencing psychological aspects including self-confidence, self-esteem, or motivation [23]. In that sense, HRS is supposed to produce three-dimensional movements similar to the walking patterns of a live animal [30]. Based on the hippotherapy research literature, during riding, the patient’s pelvis moves in a soft, rhythmic, and repetitive pattern, being a movement similar to that performed by our pelvis during normal human walking. Repetitive movements improve postural coordination and rhythm and allow reciprocal movement, in addition to facilitating postural control through stimulation of balance reactions [23] and adaptive behaviors and movement strategies, due to the changing environment in which the session takes place [31]. Maintaining the center of gravity within the support base while the animal is walking means that the patient has to anticipate and compensate for postural adjustments by reducing the center of gravity in order to remain safe on a moving surface such as the rump of the horse, stimulating multiple sensory inputs and efferent outputs [30], providing continuous motor, visual, somatosensory, and vestibular inputs to the patient [25].

There is controversy in this regard, as some therapists suggest that these devices only mimic the mechanical pattern of movement and lose all psychological aspects related to hippotherapy, as well as the heat the horse transmits [29]. Although, in favor of the HRS, it generates between 50 and 100 three-dimensional physical movements (forward and back, left and right, up and down) [32]. Some examples of HRS devices are JOBA [32], OSIM uGallop, Taiwan [30], Fortis [33], S-RIDER Shinwha Electron [34], Honjin, Korea [25], and Model H-702 [35], HJLCo. Ltd., Seoul Korea [31]. HRS have been used to produce benefits on chronic low-back pain (improvements in pain, trunk strength, muscle mass, and balance) [31]; on activation of trunk and thigh muscles, improved postural control, and balance in older adults [33], as well as in ambulation, prevention of fall risk, and improvement of dynamic stability [25,35]; on balance and gait cadence in healthy subjects [32]. Nevertheless, to our knowledge, there is only a clinical trial comparing the effect of the hippotherapy versus the HRS in neurological rehabilitation [30]. It reported that hippotherapy is the most effective intervention for improving sitting abilities, measured by the Gross Motor Function Measure (GMFM) Sitting Dimension. Thus, it is difficult to analyze and compare the effects of the two techniques due to the limited evidence.

In this view, our main objective is to analyze the effectiveness of the use of HRS on the improvement of postural balance in subjects with neurological disorders, since the current evidence contrasted through systematic review in this subject is limited. Furthermore, this study aims to provide a global overview of the evidence-based practice of HRS in neurological rehabilitation and facilitate the design of new lines of research on this topic.

## 2. Materials and Methods

This review has been conducted following the Preferred Items for Systematic Reviews and Meta-Analyses (PRISMA) statement [36].

### 2.1. Search Strategy

The literature search for the present review was conducted during the months of January–March 2019, using the following databases: PubMed, Physiotherapy Evidence Database (PEDro), The Cochrane Library, Web of Science (WoS), CINAHL, and Scopus. The search details are shown in Table 1. In the case of Pubmed, Medical Subjects Headings (MeSH) descriptors were used. No filters were applied as to the date of publication, nor the type of studies in the database. Instead, the English language filter was used.

### 2.2. Eligibility Criteria

Studies included in this review met the following inclusion criteria: (1) Participants were children and adults with neurological disorders; (2) HRS-based interventions compared with non-intervention, placebo or conventional physical therapy (CPT); (3) outcome: postural balance; (4) study design: randomized and nonrandomized controlled trials. Studies were excluded from this review if the sample included healthy subjects or people with different disorders, but the outcome data were not shown separately for participants.

### 2.3. Selection Process and Data Extraction

Firstly, the search was performed by combining the keywords previously described in the different databases. Potentially relevant articles were identified after reading the title and abstract, and duplicated articles were eliminated. Subsequently, an exhaustive verification of compliance with the inclusion criteria was carried out to obtain the articles included in the systematic review.

Two reviewers (J.G.D.R. and A.M.A.) were actively involved in the process of study selection, review, and systematic extraction of data from each included study. An additional reviewer (D.L.A.) has participated in the consensus of the different decisions. The following information has been extracted from each article included in the systematic review: author, year of publication, characteristics of the participants (total number of participants, number of participants in both groups, average age), and characteristics of the intervention carried out (type, frequency, duration of the session, outcome measures, measurement instruments, and results).

### 2.4. Assessment of the Methodological Quality of the Studies

The PEDro [37] scale was used to assess the methodological quality of the studies included in this review. It consists of 11 items related to the domains of selection, performance, detection, information, and attribution bases [38]. Taking into account the established criteria, a study with a PEDro score of 6 or more would be considered of good methodological quality (6–8 good; 9–10 excellent); and a study with a score of 5 or less would be considered of poor methodological quality (4–5 acceptable), ≤4 poor) [39].

### 2.5. Statistical Analysis

A meta-analysis was carried out to compare changes in the effect size (pre- and post-intervention) between the intervention group and the comparison group. The studies were grouped according to the outcome measure. For each meta-analysis, the standardized mean difference was calculated, along with the 95% confidence interval (CI). The significance level was set at *p* < 0.05. Heterogeneity was determined by the chi-square test and the I2 statistic. When homogeneity was observed, a fixed-effect model was used. In the case of heterogeneity, a random-effects model was used. All statistical analyses were carried out by using the statistical software Review Manager (RevMan) 5.3 (The Cochrane Collaboration, The Nordic Cochrane Centre, London, UK). The results are presented in Forest plots.

## 3. Results

**Figure 1:**
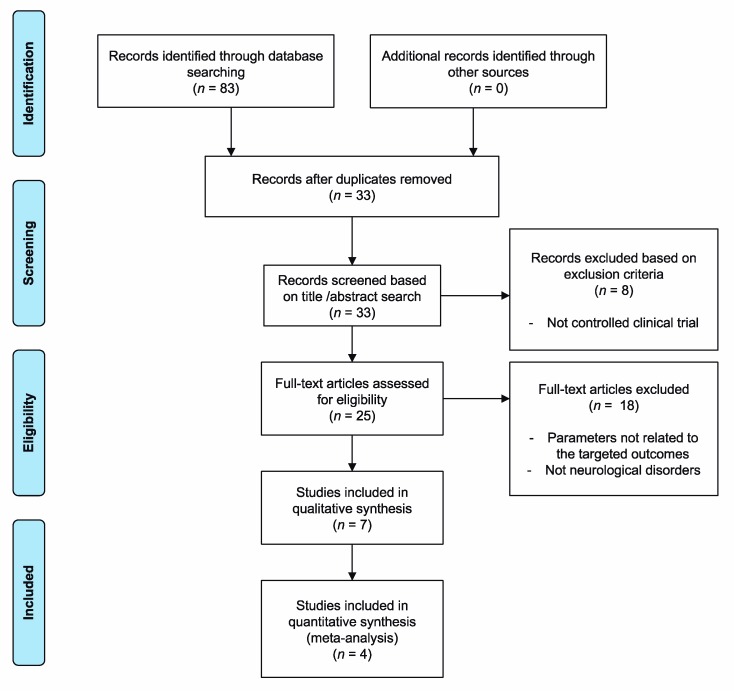
Information flowchart of the different phases of the systematic review and meta-analysis.

Firstly, a total of 83 articles were obtained. Once the duplicates were removed, 33 articles were obtained. After the selection process, a total of seven studies were included in the review, of which four were included in the meta-analysis for the statistical comparison. The entire selection process in the corresponding phases is detailed in Figure 1.

### 3.1. Assessment of the Methodological Quality of the Studies

The methodological quality of the studies included in this review was generally acceptable (average total PEDro score = 4.4, range 3–8). Five studies [29,30,40,41,42] were randomized clinical trials. The study with the highest score on the PEDro scale was that of Herrero et al. [29] with a score of 8, considering good its methodological quality. On the other hand, three studies [41,43,44] obtained the lowest score with a score of 3, considering poor its methodological quality. Table 2 shows the results obtained by the different studies included in the PEDro scale.

### 3.2. Characteristics of Participants

The mean ages of the participants ranged from 5 to 62 years. Borges et al. [40] included the youngest sample, and Han et al. [43] the oldest sample. Concerning gender distribution, there are two balanced studies [30,44], three with a higher proportion of men [29,41,43], and one with a higher proportion of women [40]. Regarding the sample size, Park et al. [44] had the highest total number of participants (*N* = 67); Choi et al. [41], Cho et al. [42], and Temcharoensuk et al. [30] had the lowest sample size (*N* = 30). Furthermore, there is no information about the sample size calculation, and in some cases, the selected number of subjects could be insufficient, becoming a design drawback. The average number of participants among the different studies is approximately 39 participants. Moreover, to know the homogeneity between groups, the disability degree of the subjects with CP has been addressed using the Gross Motor Function Classification System (GMFCS) and the time after onset disease was considered for stroke patients. The main characteristics of the participants are shown in Table 3.

### 3.3. Characteristics of the Interventions

In terms of comparison intervention, five studies [40,41,42,43,44] compared the effects of HRS with CPT. Exercises performed on a mattress in the study by Park et al. [44] have been considered CPT. Herrero et al. [29] and Temcharoensuk et al. [30] used the off HRS as a control group. The study with the longest total duration of intervention was that of Han et al. [43] with a duration of 12 weeks. The one with the shortest intervention time was Temcharoensuk et al. [30], who only performed one treatment session. Considering the target population on which the studies were conducted, four studies [29,30,40,41] analyzed the effects of HRS intervention in subjects with CP, while three other studies [42,43,44] analyzed the effects on subjects with stroke. Concerning the different HRS devices, they differ in the structure and type of programming they present, varying in directions of movement, numbers of speeds, and the inclusion or not of pre-established programs. The most commonly used devices in the included studies were JOBA [40,41,43] and FORTIS [42,44]. Table 4 presents the main characteristics of the interventions and the results obtained.

The characteristics of the different groups created for the meta-analysis are shown in Table 5. The neurological disorder and the outcome measure were considered for the creation of these groups. Accordingly, two groups were established: (i) Berg Balance Scale in stroke patients and (ii) GMFM-66 Sitting Dimension in patients with CP.

**Figure 2:**
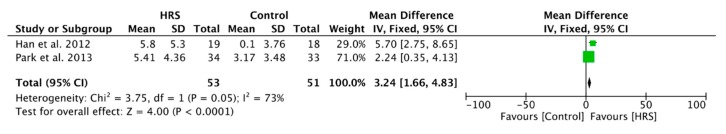
Forest plot for BBS in stroke patients.

**Figure 3:**
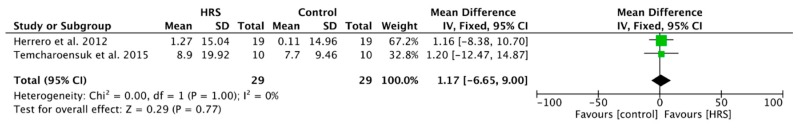
Forest plot for GMFM-66 Sitting Dimension in patients with cerebral palsy.

Firstly, analyzing the effects on balance measured employing Berg Balance Scale (BBS), the results showed that HRS caused significant improvements compared with the control group, which received CPT. The study of Han et al. [43] was the one that had a major effect on weight. The overall result of the meta-analysis is favorable. Regarding the effects of sitting postural balance measured using Gross Motor Function Measure (GMFM-66) Sitting Dimension, none of the interventions produced a significant improvement. The overall result of this meta-analysis is not conclusive. Figure 2 and Figure 3 show the results obtained after the meta-analysis.

## 4. Discussion

It is important to remark that, to the best of our knowledge, this is the first meta-analysis summarizing the findings on HRS interventions to improve postural balance in neurological rehabilitation in the literature. A total of seven articles were reviewed which analyzed the application of HRS in patients with stroke and CP, using a diversity of instruments and scales for the evaluation of postural balance.

CP and stroke usually produce alterations in the postural adaptation along with an impairment of balance and posture. Abnormal input of sensory information greatly influences postural oscillation and muscle activity, interfering with treatment interventions, causing disability in activities of daily living and difficulty in achieving independent individual performance [40,44]. Making a global consideration about the postural balance deficit management, there is evidence of the use of HRS in different population groups, such as in young people [33], chronic low-back pain [31] and elderly [25,35], such as stated in the introduction section. These results could not be extrapolated to our study population since they present different clinical characteristics. If we assume the similarity between HRS and hippotherapy interventions, the present review highlights the benefits of HRS therapy on postural balance in subjects with CP and stroke, coinciding with the results obtained by Stergiu et al. [45] in their review of the effects of hippotherapy on balance disorders. In that way, some comments and considerations about the articles included in the meta-analysis and systematic review need to be addressed.

First of all, there are some characteristics related to the studies’ design that could be influencing the results obtained. Considering the risk of bias, assessed by the PEDro scale, only two studies [29,30] have good quality (score > 6). As a starting point, the results of the present analysis should be taken with caution. The gender disparity of the included studies could affect the outcome. We cannot assume that the benefits obtained are similar in men and women. Moreover, the disability degree is a factor not always well controlled in this type of study. Neurological patients could have heterogeneous characteristics, conforming groups of functional levels highly diverse. It is measured using scales of functionality, such as GMFCS, but sometimes the analysis of the results are considered over the whole sample. In our review, there are two studies [29,30] that use stratification for patient allocation, but only one [29] analyzes the results separately. The rest of the studies consider all the subjects together. Thus, stratified analyses will be necessary to determine if there are differences due to the gender physiology or baseline functional level. To do so, the sample size has to be higher to analyze an adequate number of subjects in each group. This drawback is related to the other point of concern; in general, the sample included in each group could be insufficient to obtain adequate outcomes. Nonetheless, these subjects are treated in a real clinical scenario in conjunction with their prescribed treatment in a determined institution, so that it is difficult in many cases to obtain a higher number of patients. Consequently, this makes it necessary to use samples of convenience, and most biostatistical tests assume that the study sample is probabilistically representative of the population, so it can constitute possible selection biases [46]. Further multicentric studies are needed to know the real benefits of the HRS.

As for the benefits obtained on postural balance in subjects with CP, analyzed in four studies [29,30,40,41], two studies [29,30] evaluated the effects on sitting balance, measured using GMFM-66 Sitting dimension scale. After statistical analysis, it was not possible to conclude that HRS interventions are effective to improve sitting balance in cerebral palsy. On the one hand, it should be noted that the study of Herrero et al. [29] had good methodological quality with a score of 8 on the PEDro scale. It is characterized by the comparison of HRS therapy with placebo (HRS switched off) and by using less frequency of treatment (once a week) and short session duration (15 min). Despite this, significant results were obtained on sitting balance, greater in subjects who had higher levels of disability. On the other hand, the study of Temcharoensuk et al. [30] was the only study that found no significant differences between groups in trunk control and sitting balance. Borges et al. [40] and Choi et al. [41] obtained favorable effects on postural balance in terms of anteroposterior, medial–lateral displacement, trunk imbalances, inclination, and pelvic torsion. Therefore, HRS seems to be effective in improving balance in subjects with CP, but no strong conclusions can be drawn about the effectiveness of HRS therapy on sitting balance. Regarding the benefits produced by HRS in subjects with stroke, three studies [42,43,44] obtained favorable effects on balance. Han et al. [43] and Park et al. [44] found significant results in dynamic balance, analyzed using BBS scale, in favor of HRS therapy. In addition, Cho et al. [42] found significant improvements in static balance through HRS intervention compared with CPT. Therefore, the results obtained in our study suggest that HRS turned out to be effective to improve dynamic balance in adults with stroke.

Comparison of HRS and CPT has been evaluated in five studies [40,41,42,43,44]. In these studies, the results were significant in favor of HRS therapy. Han et al. [43] and Choi et al. [41] analyzed the effects of HRS in addition to CPT, but used different intervention times: 20 min of HRS plus 30 min of CPT; and 30 min of HRS and 15 min of CPT, respectively. Both studies found significant results on balance, being the study of Han et al. [43] the one that got the most significant effects. On the other hand, Borges et al. [40], Park et al. [44], and Cho et al. [42] compared HRS versus CPT. The main difference is the session duration. Borges et al. [40] applied 40 min of HRS and Park et al. [44] applied 35 min of HRS; both studies found significant results. On the other hand, Cho et al. [42] performed 20 min of HRS, and they did not obtain favorable effects, so it is likely that the time of intervention has influenced the achievement of their results. Finally, our results suggest that HRS devices appear to be more effective than CPT in improving balance in neurological rehabilitation.

The results obtained in our study could be useful in clinical practice by using HRS devices to improve postural balance. However, the results should be taken with caution due to the limited number of studies analyzed, besides using different HRS devices, combined or not to CPT. It would be convenient to conduct randomized controlled clinical trials with higher methodological quality, using larger sample sizes, unifying protocols regarding the device used, intervention times and duration of the treatment program, measuring instruments used, and monitoring the effects on participants. It could help to know which factors of the therapy are the ones that have more impact on the positive results of the intervention. Furthermore, it is important to consider the possible inter-examiner biases that the analysis of results reported by different research groups could have. For example, Wood and Rosenbaum [47] reported interrater reliability for GMFCS of generalizability coefficient (G) = 0.93 and test–retest reliability of G = 0.79 in a group of children attended in a southern Ontario regional children’s rehabilitation center. These results were obtained in an environment in which the language and culture are similar; some other factors can affect the results when scales are used in other languages and different scenarios.

## 5. Conclusions

There is a lack of high-quality published articles assessing the effects of HRS interventions on postural balance in subjects with neurological disorders, being the main disorders analyzed CP and stroke. In that way, the present review and meta-analysis constitute a starting point for the development of new well-based research. According to the results obtained, it might be concluded that HRS devices seem to be more effective than CPT for the improvement of dynamic balance after stroke. In addition, interventions based on HRS have a positive effect on postural balance in subjects with cerebral palsy, but the evidence of improvement of sitting balance was inconclusive.

Further research into the effects of this therapy on neurological rehabilitation will be needed to use this therapy as a complement to the CPT.

## Figures and Tables

**Table 1:** Search strategy.

Databases	Total Articles	Search
PubMed	15	(“Horseback riding simulator” OR “Mechanical horse” OR “Horse simulator” OR “Horse riding simulator” OR “Hippotherapy simulator”) AND (“Postural balance” OR “Balance” OR “Equilibrium” OR “Postural control”)
PEDro	5	
Cochrane	7	
Web of Science	27	
CINAHL	12	
Scopus	17	

**Table 2:** Physiotherapy Evidence Database (PEDro) scale score for clinical trials included in the review.

PEDro Scale													
Studies	Total Score	Methodological Quality	1	2	3	4	5	6	7	8	9	10	11
Borges et al. (2011) [40]	4	Fair	-	X		X			X			X	
Herrero et al. (2012) [29]	8	Good	-	X	X	X			X	X	X	X	X
Han et al. (2012) [43]	3	Poor	-			X					X	X	
Park et al. (2013) [44]	3	Poor	-			X						X	X
Choi et al. (2014) [41]	3	Poor	-	X		X						X	
Cho et al. (2015) [42]	4	Fair		X		X						X	X
Temcharoensuk et al. (2015) [30]	6	Good	-	X		X			X	X		X	X

The ‘X’ symbol indicates that the item where it is found has been punctuated.

**Table 3:** Main characteristics of participants.

Study	Disease	Mean Age (Years ± SD)	Average Weight (Kg ± SD)	Average Height (cm ± SD)	Female:Male	Participants (*n*)	Disability Degree
Borges et al. (2011) [40]	CP	IG: 5 ± 2.48 CG: 5 ± 2.2	ND	ND	IG: 12:8 CG: 11:9	40	GMFCS Levels III-V
Herrero et al. (2012) [29]	CP	IG: 9.95 ± 5.22 CG: 9.05 ± 6.69	ND	ND	IG: 5:14 CG: 9:10	38	GMFCS Levels I-V
J. Han et al. (2012) [43]	Stroke	IG: 61.1 ± 6.3 CG: 62.2 ± 6.9	ND	ND	IG: 6:13 CG: 7:11	37	Chronic phase > 6 months
Park et al. (2013) [44]	Stroke	IG: 56.09 ± 7.22 CG: 51.55 ± 8.27	IG: 62.91 ± 7.93 CG:67.05 ± 8.38	IG: 166.05 ± 7.47 CG: 169.55 ± 8.40	IG: 16:18 CG: 15:18	67	Chronic phase > 6 months
Temcharoensuk et al. (2015) [30]	CP	IG: 10.1 ± 1.7 CG: 10.4 ± 1.5	ND	ND	IG: 4:6 CG: 5:5	30	GMFCS Levels II-III
Choi et al. (2014) [41]	CP	IG: 9.3 ± 3.8 CG: 8.8 ± 3.1	IG: 26.3 ± 9.2 CG: 26.2 ± 9.7	IG: 124.7 ± 15.8 CG: 119.6 ± 21.1	IG: 4: 11 CG: 5:10	30	GMFCS Levels I-IV
Cho et al. (2015) [42]	Stroke	IG: 54.20 ± 9.21 CG:54.00 ± 8.79	IG:62.21 ± 7.88 CG:62.31 ± 7.22	IG:163.67 ± 10.17 CG:165.23 ± 8.48	ND	30	Chronic phase > 6 months

IG: Intervention Group; CG: Control Group; SD: Standard Deviation; ND: Not Described; CP: Cerebral Palsy; GMFCS: Gross Motor Function Classification System.

**Table 4:** Main characteristics of the interventions.

Author	Condition	No. Patients	Intervention	Frequency	Session Duration	Intervention Duration	Outcome Measures	Measuring Instruments	Results
Borges et al. (2011) [40]	CP	N: 40 IG: 20 CG: 20	IG: HRS (JOBA) CG: CPT	2 times/week	40 min	6 weeks	Sitting balance	F-Mat sensor stabilometric platform (model 3100, Tecksan, Inc, South Boston, MA). Gross Motor Function Classification System.	A significant improvement in the anteroposterior (*p* < 0.0001) and medial–lateral (*p* < 0.0069) direction displacement was found in the IG compared with CG
Herrero et al. (2012) [29]	CP	N: 38 IG: 19 CG: 19	IG: HRS (ON) CG: HRS (OFF)	1 times/week	15 min	10 weeks	Sitting balance	Gross Motor Function Measure-66 (GMFM-66) Sitting Dimension. GMFM-66 Total score. Sitting Assessment Scale (SAS).	A significant improvement in sitting balance was found in the IG, measured with the GMFM-66 Sitting dimension (Effect size = 0.36). In the severe disability group, the effect size was more significant (Effect size = 0.80) The changes in GMFM-66 Total score and SAS were not significant
Han et al. (2012) [43]	STROKE	N: 37 IG: 19 CG: 18	IG: CPT + HRS (JOBA) CG: CPT	2 times/week	20 min of HRS + 30 min of CPT	12 weeks	Balance	Berg Balance Scale (BBS). B-POMA (Balance section in Performance Oriented Mobility Assessment).	Between groups, a significant improvement was obtained in dynamic balance in IG, measured using BBS (*p* = 0.02) The balance parameters, measured with BBS and B-POMA, improved significantly in the IG (*p* = 0.001) B-POMA significantly improved in IG (*p* = 0.001)
Park et al. (2013) [44]	STROKE	N: 67 IG:34 CG:33	IG: HRS (FORTIS) CG: CPT (Exercises on the mattress)	3 times/week	35 min	8 weeks	Static balance (open and closed eyes). Dynamic balance.	Kinesthetic Ability Trainer (KAT) Balance system. Berg Balance Scale (BBS).	There was a significant improvement in open and closed eyes tests measured with KAT, and a significant increase in dynamic balance, measured with BBS (*p* < 0.05) in both groups Between groups, a significant improvement was found in static balance and dynamic balance in IG (*p* < 0.05)
Temcharoensuk et al. (2015) [30]	CP	N: 30 IG:10 CG: 10 IG2: 10	IG: HRS (ON) (OSIM uGallop, Taiwan) CG: HRS (OFF) (OSIM uGallop, Taiwan)	1 session	30 min	1 session	Trunk control. Sitting balance.	Segmental Assessment of Trunk Control (SATCo). Gross Motor Function Measure (GMFM-66) Sitting Dimension.	In SATCo pre–post intervention, the ON HRS group showed differences in active (*p* = 0.034) and reactive trunk control (*p* = 0.034) scores; and the OFF HRS group showed differences only in the active trunk control score (*p* = 0.046). No differences were found between groups. No significant differences were found in the GMFM-66 Sitting Dimension between groups.
Choi et al. (2014) [41]	CP	N:30 IG:15 CG:15	IG: CPT + HRS (JOBA) CG: CPT	4 times/week	IG: 30 min of CPT + 15 min of HRS CG: 30 min of CPT	10 weeks	Trunk imbalance, pelvic torsion and pelvic tilt	Spinal structure analysis system: ABW Mapper	Significant effects on trunk imbalance, pelvic torsion and pelvic tilt were found in the IG compared with the CG (*p* < 0.05)
Cho et al. (2015) [42]	STROKE	N: 30 IG: 15 CG: 15	IG: HRS (FORTIS) CG: CPT	IG: CPT 5 times/week + HRS 3 times/week CG: CPT 5 times/week	IG: 30 min of CPT + 20 min of HRS CG: 30 min CPT	6 weeks	Static balance under standing position	Romberg test using Balance measuring equipment: BIORescue (Force plate to sense moving distance of the center of pressure).	There were no significant differences between groups (*p* < 0.05). There was a significant improvement in moving distance under standing position with eyes closed in the IG group (*p* < 0.05).

CP: Cerebral Palsy; N: Number; IG: Intervention Group; CG: Control Group; CPT: Conventional Physical Therapy; HRS: Horse-Riding Simulators.3.4. Groups and subgroups included in the meta-analysis.

**Table 5:** Study groups included in the Meta-analysis.

Group	Studies	Outcome Measure	Neurological Disorder
1	Han et al. [43], Park et al. [44]	Berg Balance Scale	Stroke
2	Herrero et al. [29], Temcharoensuk et al. [30]	Gross Motor Function Measure-66 Sitting Dimension	Cerebral palsy
